# Supplementary material for: Tandem Mass Spectrometry as Strategy for the Selective Identification and Quantification of the Amyloid Precursor Protein Tyr682 Residue Phosphorylation Status in Human Blood Mononuclear Cells
Source: Biomolecules. 2021 Aug 31;11(9):1297. doi: 10.3390/biom11091297 (PMC8471626; doi:10.3390/biom11091297)

Supplementary Figures

Supplementary Figure S1. HELA cells express GFP tagged APP and apple tagged Fyn 48hrs after transfection. (**a**) APP and Fyn WB analysis in transfected (APP + Fyn) and untransfected cells (CTRL). APP-GFP and Fyn-apple migrate approximately at 150kDa and 85kDa, respectively. (**b**) WB analysis of APPpTyr in transfected and untransfected cells.

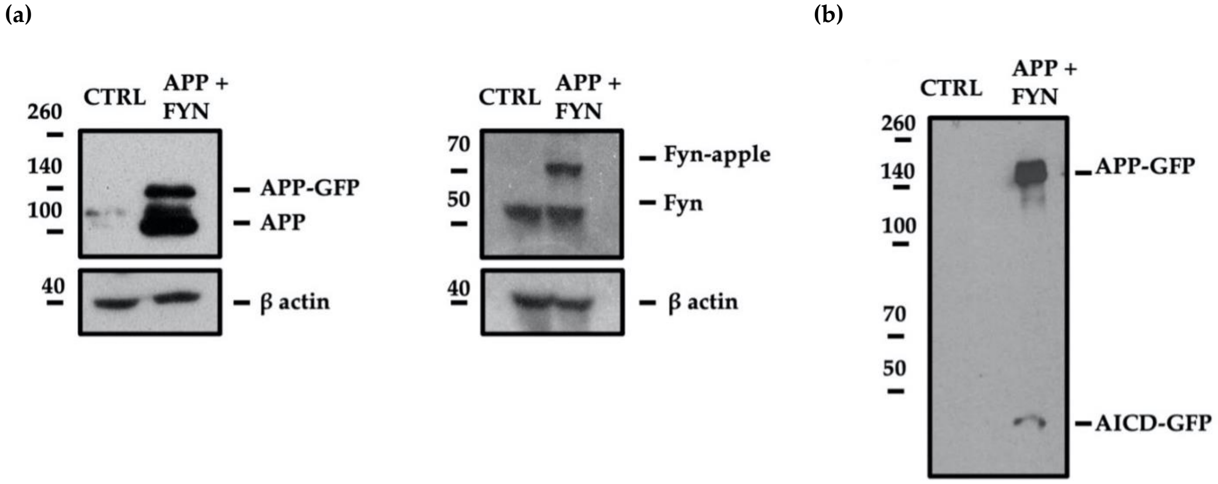

Supplementary Figure S2. Mass spectra of G.A patient: (**A**) Extract ion chromatograms of MQQNGYpENPTYK, (**B**) MRM transition 776.8 > 508.1 and 776.8 > 1050.8, (**C**) Enhanced product ion spectrum of MQQNGYpENPTYK.

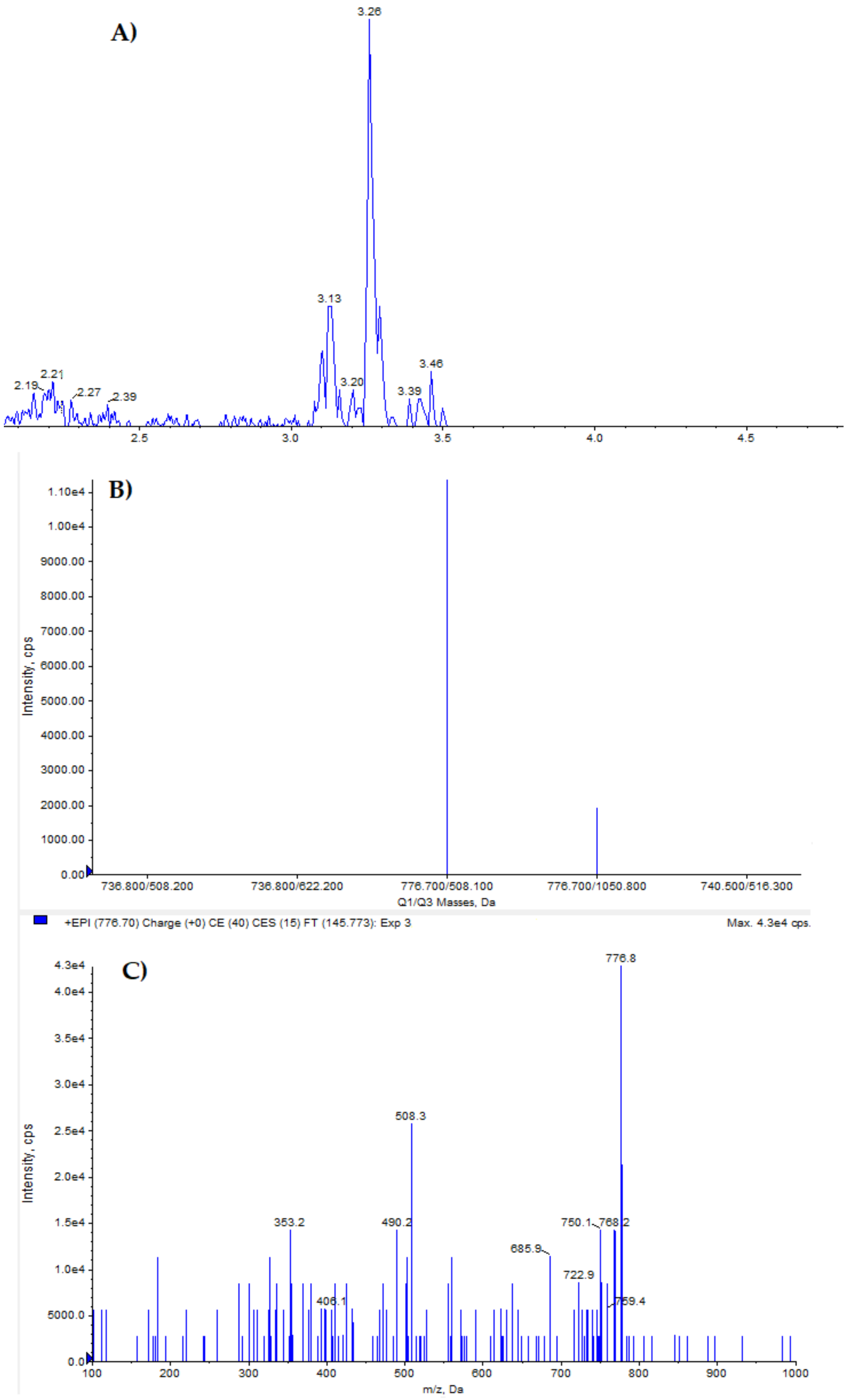

Supplementary Figure S3. Mass spectra of O.S. patient: (**A**) Extract ion chromatogram of MQQNGYpENPTYK, (**B**) MRM transition 776.8 > 508.1 and 776.8 > 1050.8, (**C**) Enhanced product ion spectrum of MQQNGYpENPTYK, (**D**) Extract ion chromatogram of MQQNGYENPTYK, (**E**) MRM transition 736.8 > 508.2 and 736.8 > 622.2, (**F**) Enhanced product ion spectrum of MQQNGYENPTYK.

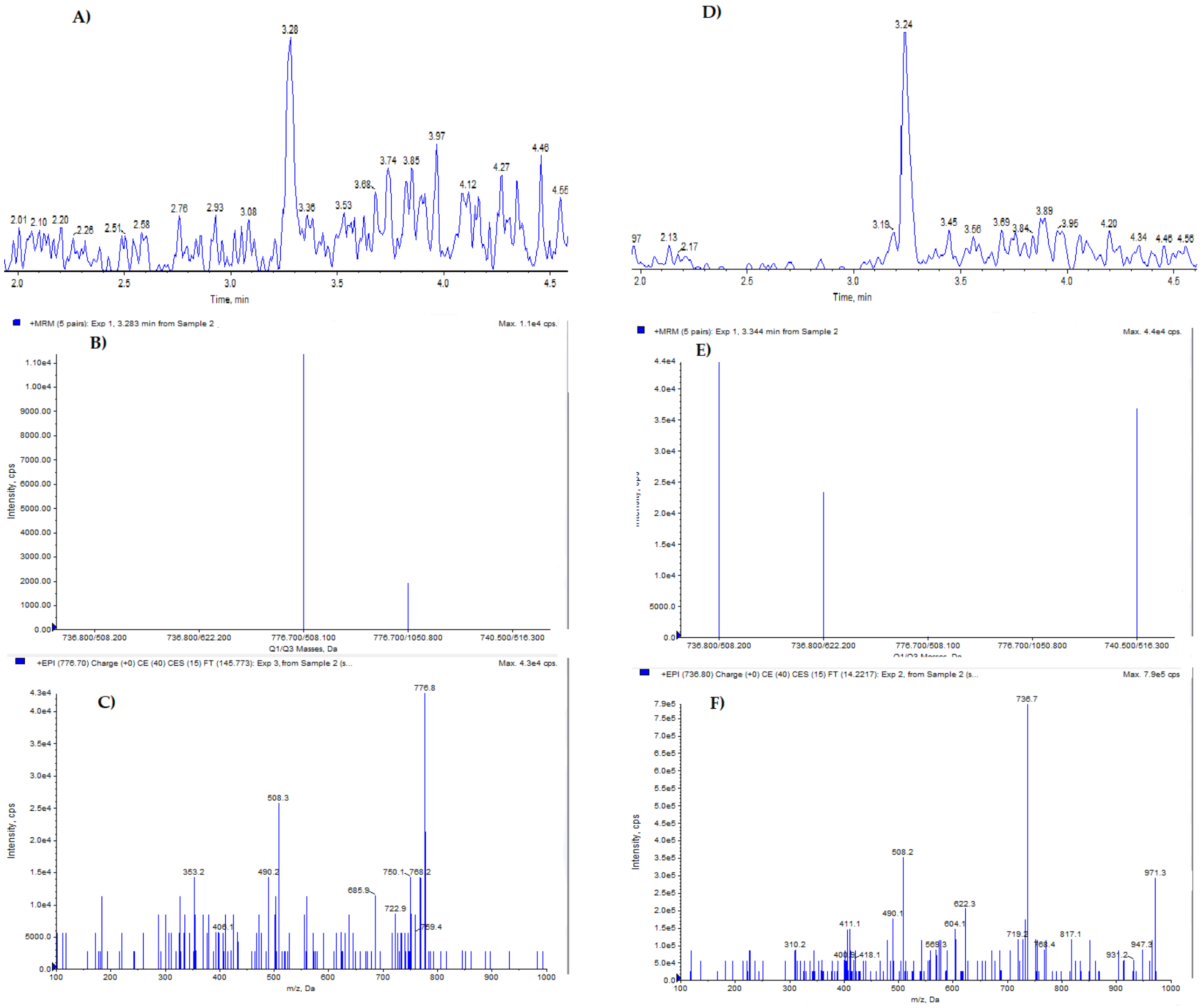

Supplement: Supplementary file 1 [file biomolecules-11-01297-s001.zip › Supplementary figures.pdf]
