# Supplementary figures and images for: Tandem Mass Spectrometry as Strategy for the Selective Identification and Quantification of the Amyloid Precursor Protein Tyr682 Residue Phosphorylation Status in Human Blood Mononuclear Cells
Source: Biomolecules. 2021 Aug 31;11(9):1297. doi: 10.3390/biom11091297 (PMC8471626; doi:10.3390/biom11091297)

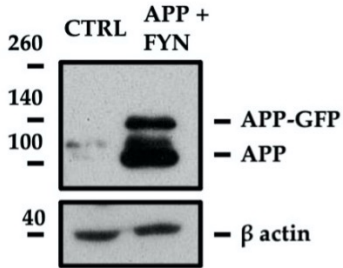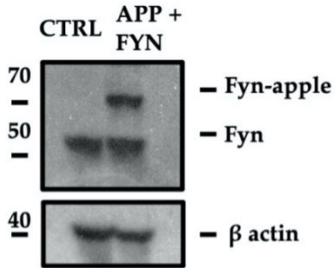

Supplement: Supplementary file 1 [file biomolecules-11-01297-s001.zip › Supplementary Figure S1a-Biomolecules.pdf]

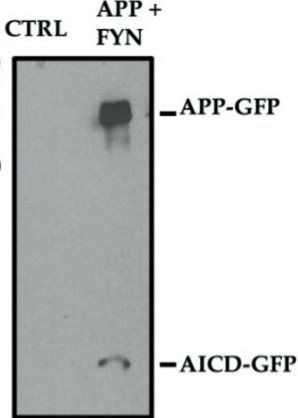

Supplement: Supplementary file 1 [file biomolecules-11-01297-s001.zip › Supplementary Figure S1b-Biomolecules.pdf]

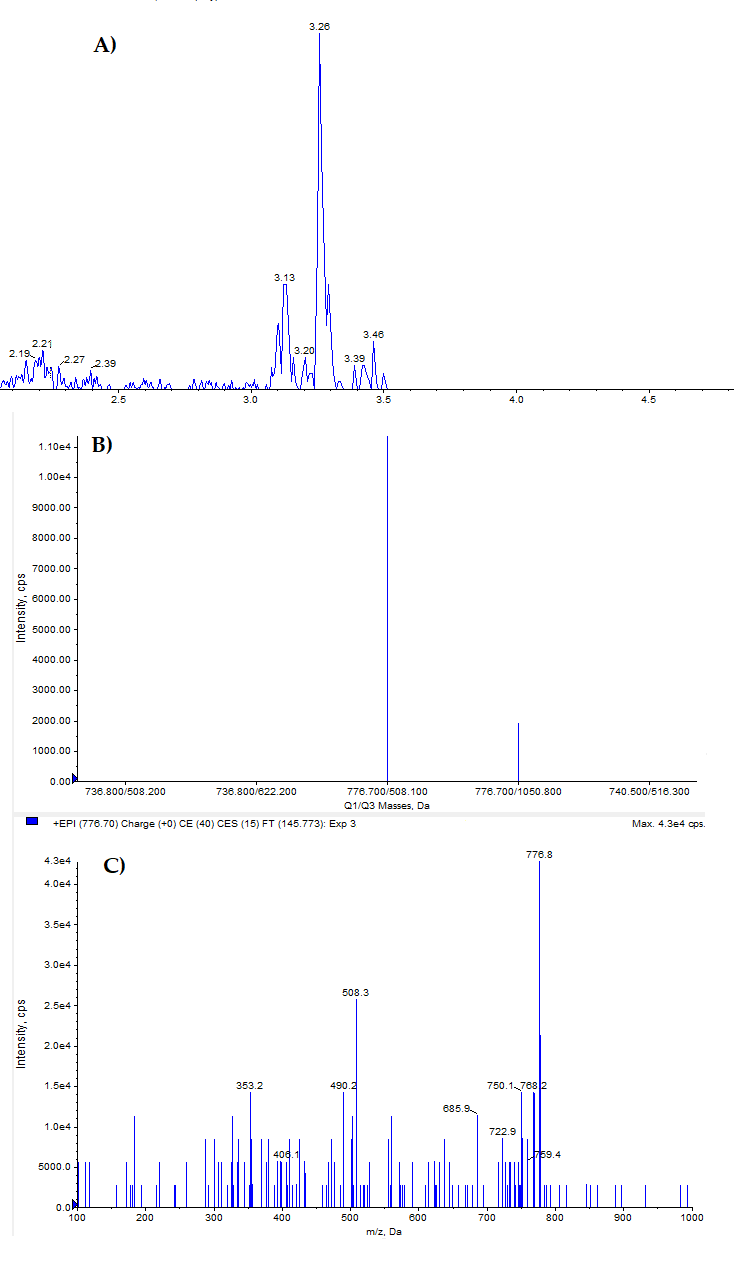

Supplement: Supplementary file 1 [file biomolecules-11-01297-s001.zip › Supplementary Figure S2.tif]

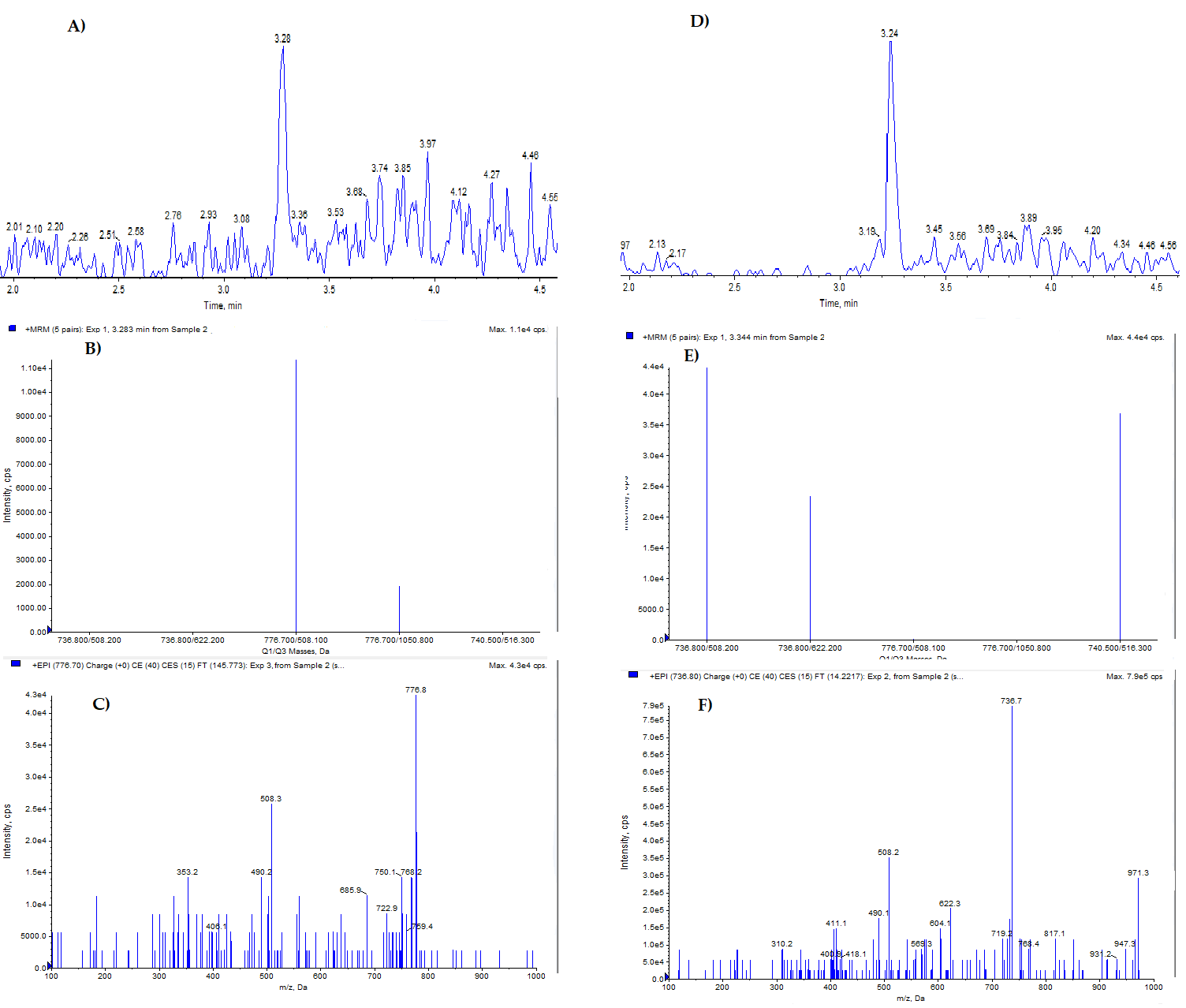

Supplement: Supplementary file 1 [file biomolecules-11-01297-s001.zip › Supplementary Figure S3.tif]
